# A Case of Early-Stage Ovarian Carcinoid Tumor Metastasized to the Liver

**DOI:** 10.1155/2012/961087

**Published:** 2012-12-26

**Authors:** Seiji Kanayama, Yoshihiko Yamada, Yasuhito Tanase, Shoji Haruta, Akira Nagai, Ryuji Kawaguchi, Shozo Yoshida, Naoto Furukawa, Hidekazu Oi, Hiroshi Kobayashi

**Affiliations:** Department of Obstetrics and Gynecology, Nara Medical University, 840 Shijo-cho, Nara, Kashihara 634-8522, Japan

## Abstract

We report a case of ovarian carcinoid tumor that recurred with multiple liver metastases and was successfully treated with chemoembolization. A 76-year-old woman was admitted to our hospital presented with abdominal distension and abnormal uterine bleeding for about 6 months. She presented with hyperestrogenic and androgenic manifestations such as vaginal bleeding with endometrial hyperplasia and hirsutism. Magnetic resonance (MR) imaging revealed a large solid and cystic ovarian tumor of 17 cm at maximum diameter. On the basis of the clinical diagnosis of sex cord stromal tumor containing a mature cystic teratoma, she underwent total abdominal hysterectomy and bilateral salpingo-oophorectomy. The pathology report revealed that the mass in the left ovary was a carcinoid tumor, insular type, with mature cystic teratoma. Two years after surgical treatment, multiple liver metastases were revealed by abdominal CT. Hepatic arterial infusion of cisplatin was performed for 2 courses, and multiple metastatic nodules have remarkably reduced. No established chemotherapy or radiation therapy treatments are currently available for recurrent or advanced carcinoid tumors. Our paper suggests that chemoembolization with cisplatin may be effective in treatment of patients with multiple liver metastases of ovarian carcinoid tumor.

## 1. Introduction

Primary ovarian carcinoid tumors are rare neoplasms. Carcinoid tumors mostly occur in the gastrointestinal tract and the lungs [1]. Ovarian carcinoid tumors constitute only 0.5% of all carcinoid tumors and <0.1% of all ovarian carcinomas [2]. We encountered a case of stage I primary ovarian carcinoid tumor with hyperestrogenic and androgenic manifestations such as vaginal bleeding with thick endometrium and hirsutism. Although some types of ovarian tumors have been reported to show androgenic clinical manifestations, ovarian carcinoid tumors with virilization are extremely rare. Most ovarian carcinoids are in the early stage and are usually curable with surgery alone [3, 4]. Here, we report for the first time a case of ovarian carcinoid tumor that recurred with multiple liver metastases and was successfully treated with chemoembolization.

## 2. A Case Report

**Figure 1:**
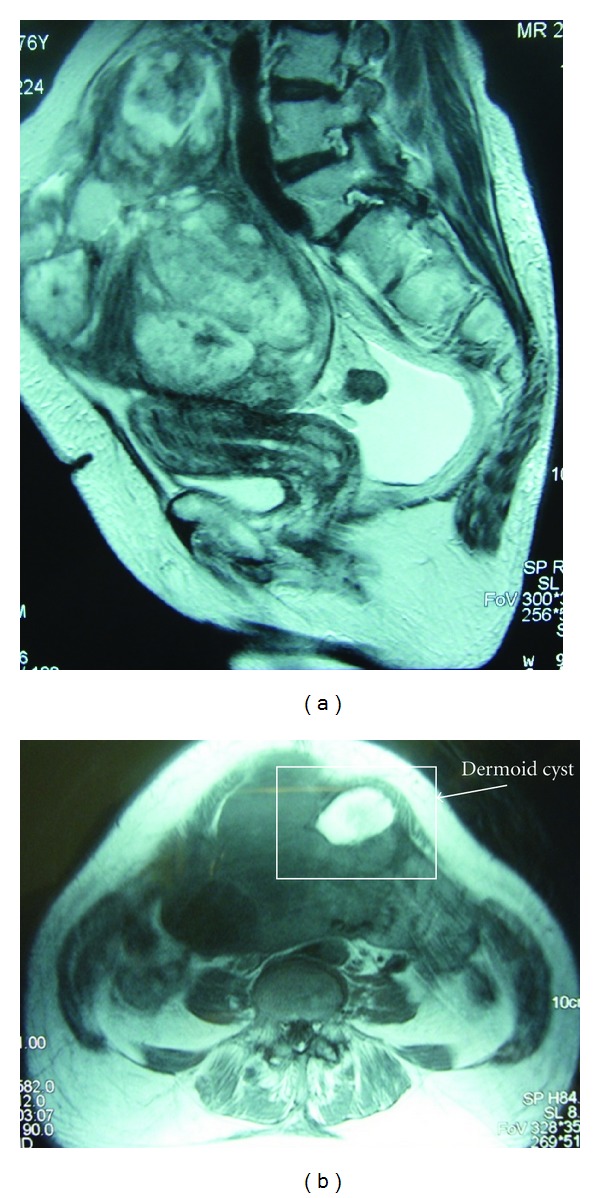
(a) Sagittal T2-weighted MR image shows a large solid and cystic ovarian tumor of 17 cm at maximum diameter and small amount of ascites. The tumor had arisen from the left ovary, and the margin was smooth. Uterus was normal size but accompanied with slightly thickened endometrium and distinct zonal anatomy. (b) Axial T1-weighted MR image revealed a mature cystic teratoma of 4 cm in a diameter which included fat component inside the tumor at the left anterior side.

A 76-year-old woman was admitted to our hospital presenting with abdominal distension and abnormal uterine bleeding for about 6 months. Physical and pelvic examinations revealed a giant pelvic mass which was the size of adult head, hirsutism on the face, and an endocervical polyp. She had no clitorimegaly or voice deepening. Preoperative cytology of the cervix of uterus and endometrium showed no atypical cell pattern. The cervical polyp was benign, and biopsy of the postmenopausal endometrium showed endometrial hyperplasia without atypia. Laboratory data showed increased concentrations of serum testosterone and estradiol (4.32 ng/mL and 69.1 pg/mL, resp.). The serum preoperative cancer antigen (CA) 125 level was 37.1 U/mL. Magnetic resonance (MR) imaging revealed a large solid and cystic ovarian tumor of 17 cm at maximum diameter containing a mature cystic teratoma of 4 cm in diameter (Figure 1). The tumor had arisen from the left ovary, and the uterus was normal in size but has a slightly thickened endometrium. The initial imaging diagnosis was metastatic ovarian cancer derived from a gastrointestinal organ, and the secondary diagnosis was sex cord stromal tumor, such as Sertoli-Leydig or granulosa cell tumor, containing a mature cystic teratoma. Preoperatively, she underwent gastrointestinal examination such as colonoscopy and gastroscopy, which were negative for tumor. Computed tomography (CT) scanning of the chest and abdomen revealed no metastatic tumor and lymph node involvement.

**Figure 2:**
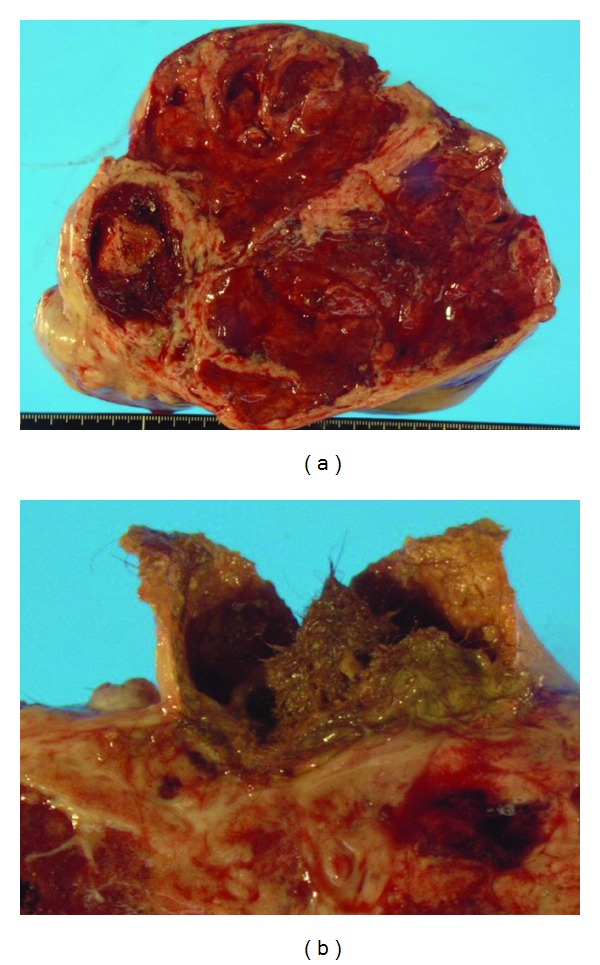
The cut surface of tumor was yellow-brown and predominantly solid with a mature cystic teratoma (4 cm at its largest diameter) containing hair ball and fat tissue.

On the basis of the clinical diagnosis of sex cord stromal tumor containing a mature cystic teratoma, she underwent total abdominal hysterectomy and bilateral salpingo-oophorectomy. Laparotomy revealed that the tumor had arisen from the left ovary and had a smooth surface; the tumor measured 19 cm at its largest diameter. The cut surface was yellow-brown and predominantly solid with a mature cystic teratoma containing hairball and fat tissue (Figures 2(a) and 2(b)). No metastatic lesion was seen in the peritoneum or omentum. 

**Figure 3:**
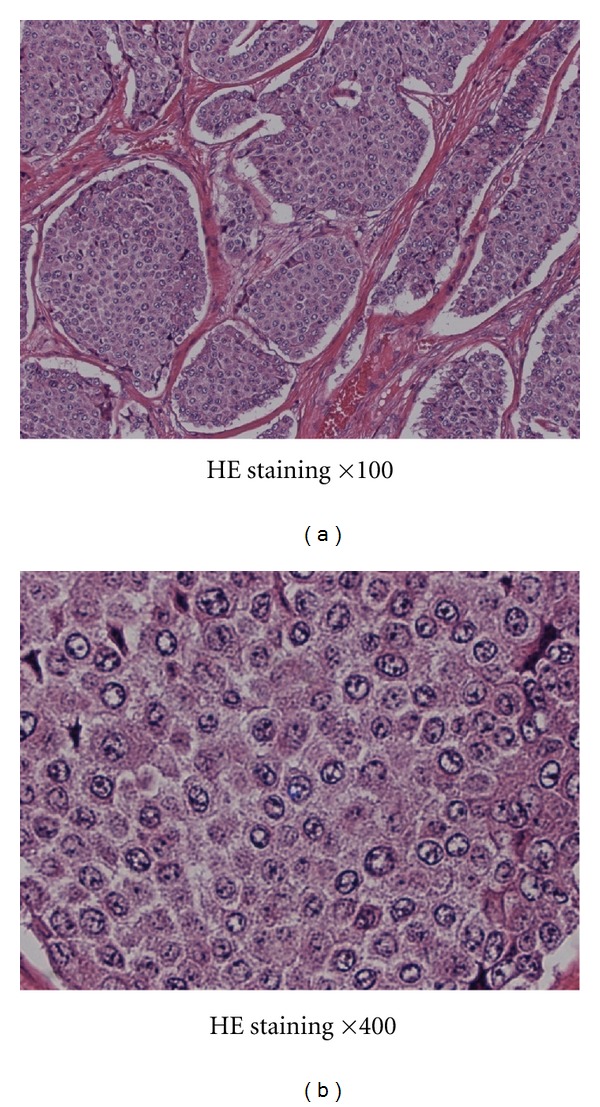
Histologic study of resected tumor by hematoxylin and eosin stain revealed that tumor composed of largely island pattern of neoplastic cells which were medium in size and had round regular nuclei with eosinophilic cytoplasm.

**Figure 4:**
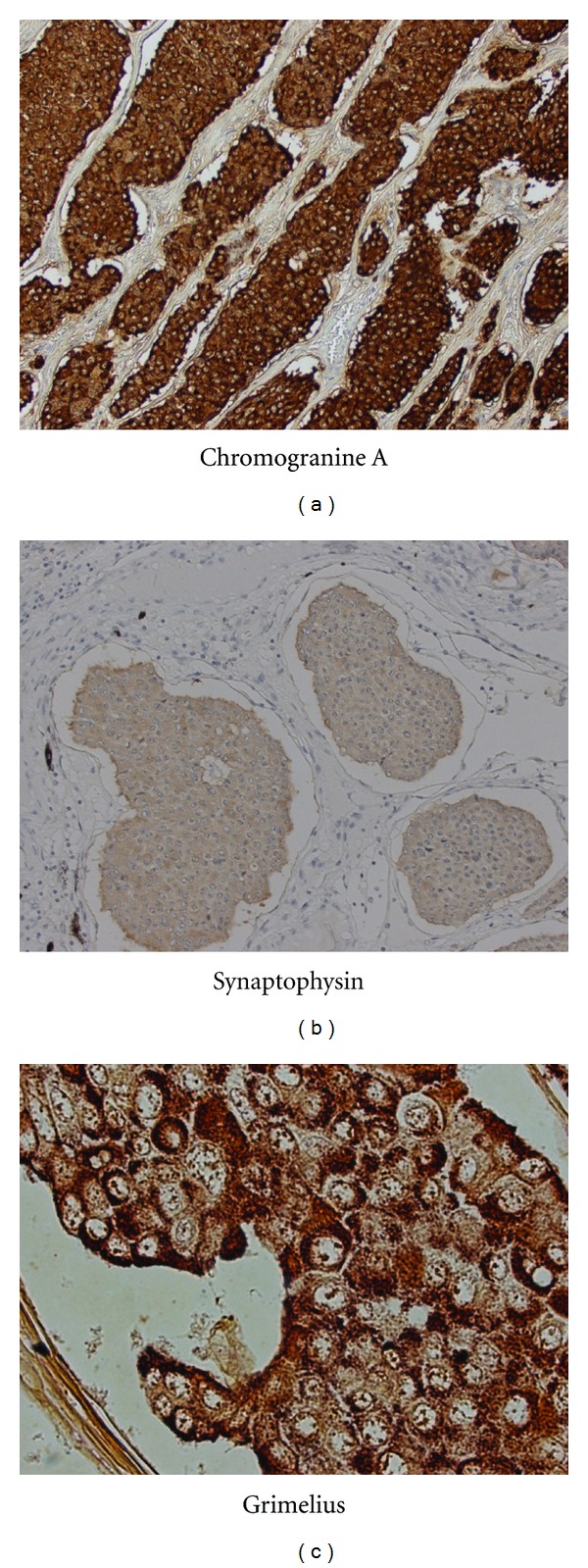
An immunohistochemical examination of the tumor cells showed positive staining for (a) chromogranin A, (b) synaptophysin, and (c) Grimelius.

**Figure 5:**
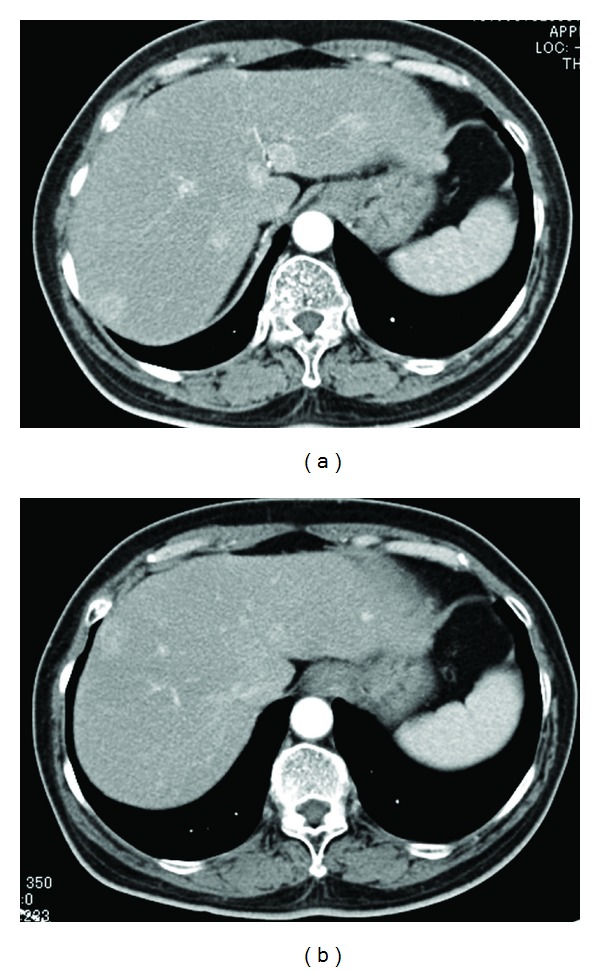
(a) Abdominal CT scanning after 24 months from surgical treatment showed multiple small nodules in the liver with no abnormal findings in other organs. (b) Multiple metastatic nodules have remarkably reduced after two cycles of hepatic arterial infusion of cisplatin.

**Figure 6:**
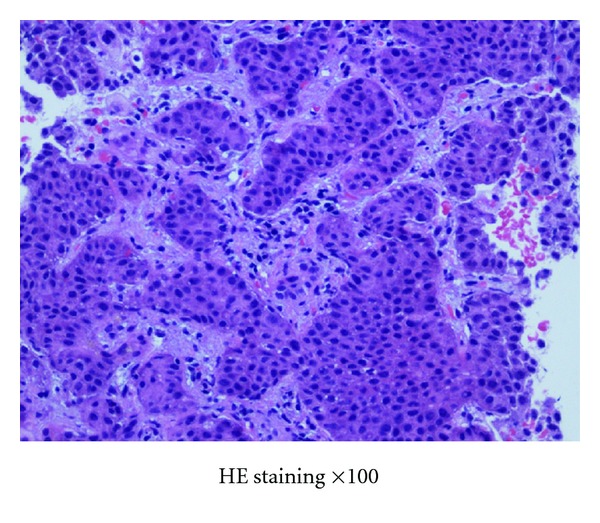
Percutaneous biopsy of the liver tumor was performed. Histological findings for the hepatic tumors were similar to the primary lesion, indicating hepatic metastasis from ovarian carcinoid tumor.

Microscopically, the tumor was composed of a largely island pattern of neoplastic cells with a focal tubular or trabecular component; the cells were medium in size and had round regular nuclei with eosinophilic cytoplasm (Figure 3). The tumor cells were surrounded by fibrous stroma and had no mitoses and necrosis. The cyst of teratoma had no immature components. Immunohistochemical examination of the tumor cells showed positive staining for Grimelius, chromogranin A, and synaptophysin (Figure 4). The pathology report revealed that the mass in the left ovary was a carcinoid tumor, insular type, with mature cystic teratoma. The International Federation of Gynecology and Obstetrics (FIGO) surgical stage was IA. She was not treated with adjuvant chemotherapy or radiotherapy. The serum estradiol and testosterone levels decreased immediately (as shown in Table 1), and hirsutism disappeared within 4 weeks after surgery. She had an uneventful postoperative course and was well 2 years after the surgery. She had no further complaints of abdominal pain or discomfort; however, routine abdominal CT scan at 24 months after surgical treatment showed multiple small nodules in the liver with no abnormal findings in other organs (Figure 5(a)). Percutaneous biopsy of the liver tumor was performed. Histological examination revealed a metastatic carcinoid tumor similar to the primary lesion (Figure 6). She received hepatic artery chemoembolization. Hepatic arterial infusion of cisplatin (40 mg/body) was performed for 2 courses until now and her multiple metastatic nodules have remarkably reduced (Figure 5(b)). She is alive with disease and residual lesion remained stable in size for about 12 months. Her performance status did not deteriorate throughout the chemotherapy period. The patient and her family were informed that the data from the medical record would be submitted for publication and gave their consent.

## 3. Discussion

Primary ovarian carcinoid tumors are rare and make up less than 0.1% of all ovarian carcinomas [2]. Ovarian carcinoid tumors are pathologically categorized into 4 groups: insular, trabecular, stromal, and mucinous types [3]. About one-third of the tumors, especially the insular types, are known to be associated with carcinoid syndromes, such as facial flushing, diarrhea, and edema, due to the direct production of serotonin-like substances into the systemic circulation through the ovarian venous system bypassing hepatic deactivation [4]. 

The ovarian tumor in our case was an insular type carcinoid with no symptoms of carcinoid syndrome. However, our case is rare for 2 reasons. First, the tumor was hormonally active, and the patient showed estrogenic and androgenic manifestations such as thick endometrium and hirsutism. Sex cord stromal tumors such as stromal Leydig tumors and Sertoli-Leydig cell tumors are well known to be associated with clinical virilization. Thecomas and granulosa cell tumors are also well known to show hormonal activity [5]. However, ovarian carcinoids exhibiting estrogenic or androgenic manifestations are extremely rare [5–7]. In the case series of Robboy and Scully, stromal carcinoids of primary ovarian origin, 3 of 50 patients had hirsutism or virilism [8]. Utsumi reported 5 cases of ovarian tumors with androgenic clinical manifestations (3 Krukenberg tumors, 1 yolk sac tumor, and 1 stromal carcinoid) [9]. As noted by Scully, neoplastic cells themselves are not primarily responsible for steroid hormone production; however, by growing within the ovarian stroma, they stimulate its cells or the adjacent hilus cells to become hormonally hyperactive [6]. Some ovarian tumors are known to have hormonal activity because they contain a functioning stroma and are capable of secreting estrogens or androgens [6, 10]. Tanaka et al. reported 2 cases of ovarian tumors with a functioning stroma: one was an atypical carcinoid and the other was a mucinous cystadenoma [11]. 

Second, our case is rare in the sense that the early-stage carcinoid tumor recurred after the initial surgical treatment. Most cases of ovarian carcinoid tumors are in the early stage and curable by excision alone [3, 4]. In Davis et al.'s case series and review of primary ovarian carcinoids, 11 of 17 patients presented with stage I disease and showed a 100% survival rate at 5 years. In contrast, 6 advanced stage patients had a 5-year survival rate of 33%, with a median survival of 1.2 years. In their case series, only 1 patient with clinical stage I disease had recurrence in the neck 14 years after the initial diagnosis [4]. 

No established chemotherapy or radiation therapy treatments are currently available for recurrent or advanced carcinoid tumors. Systemic chemotherapy has a limited role in the treatment of patients with carcinoid tumors, and only a few case reports demonstrated successful treatments with intravenous 5-fluorouracil- or cisplatin-based chemotherapy [4, 12]. Tumor recurrence is a major problem after surgical treatment. Timmins et al. reported a case of stage I ovarian carcinoid tumor recurrent in the peritoneal cavity [13]. The patient underwent exploratory laparotomy and complete resection of all visible intra-abdominal tumors, and she was clinically disease free at 38 months followup. The author reviewed and recommended complete resection of all carcinoid tumors whenever feasible, as it will provide symptomatic relief and prolonged survival. 

The liver is the most common site for carcinoid tumor metastasis; however, the standard treatment for such metastases remains controversial because of the rarity of the disease. In general, the prognosis of patients with carcinoid tumors with diffuse, unresectable liver metastasis is poor, and the 5-year survival rate of those patients ranges from 20% to 30% [14]. Some authors recommend that liver resection may not be only palliative but also may increase survival [15–17]. In our case, the patient presented with multiple unresectable liver metastases from surgical treatment. Currently, extensive intrahepatic recurrence can be treated with either embolization or chemoembolization because of the hypervascular lesions. Several reviews have suggested that hepatic artery embolization with or without intra-arterial chemotherapy is useful for controlling symptoms with diffuse liver metastasis [18, 19]. Gupta et al. reviewed 69 patients who received hepatic artery embolization or chemoembolization for multiple liver metastases from carcinoid tumors and reported that the response rate was 67% and the median overall survival time was 31 months [18]. In our case, hepatic arterial infusion of cisplatin (40 mg/body) was performed once a month, and after 2 cycles of this chemotherapy, multiple metastatic nodules have markedly reduced. 

To our knowledge, this is the first paper on an ovarian carcinoid that recurred with multiple liver metastases and was successfully treated with chemoembolization. Although no prospective randomized studies have been performed, our experience suggests that multiple extensive liver metastases from a rare ovarian carcinoid tumor can be successfully and safely treated with chemoembolization with cisplatin. 

## Figures and Tables

**Table 1:** Hormonal profile before and after the surgical treatment.

		Before	After 7 days	After 2 months
LH	(mIU/mL)	5.33	8.05	9.34
FSH	(mIU/mL)	11.22	26.68	51.94
E2	(pg/mL)	69.1	26.9	<10
Testosterone	(ng/mL)	4.32	0.58	0.44
CA125	(U/mL)	37		2

## References

[1] Robertson RG, Geiger WJ, Davis NB (2006). Carcinoid tumors. *American Family Physician*.

[2] Talerman A (1997). Germ cell tumors of the ovary. *Current Opinion in Obstetrics and Gynecology*.

[3] Robboy SJ, Norris HJ, Scully RE (1975). Insular carcinoid primary in the ovary. A clinicopathologic analysis of 48 cases. *Cancer*.

[4] Davis KP, Hartmann LK, Keeney GL, Shapiro H (1996). Primary ovarian carcinoid tumors. *Gynecologic Oncology*.

[5] Outwater EK, Wagner BJ, Mannion C, McLarney JK, Kim B (1998). From the Archives of the AFIP: sex cord-stromal and steroid cell tumors of the ovary. *Radiographics*.

[6] Scully RE (1997). Hormonally active ovarian tumors. *Verhandlungen der Deutschen Gesellschaft für Pathologie*.

[7] Young RH (1993). New and unusual aspects of ovarian germ cell tumors. *American Journal of Surgical Pathology*.

[8] Robboy SJ, Scully RE (1980). Strumal carcinoid of the ovary: an analysis of 50 cases of a distinctive tumor composed of thyroid tissue and carcinoid. *Cancer*.

[9] Utsumi N, Hayasaka T, Motoyama T (2003). Ovarian carcinoid exhibiting double function. *Pathology International*.

[10] Rutgers JL, Scully RE (1986). Functioning ovarian tumors with peripheral steroid cell proliferation: a report of twenty-four cases. *International Journal of Gynecological Pathology*.

[11] Tanaka YO, Ide Y, Nishida M (2002). Ovarian tumor with functioning stroma. *Computerized Medical Imaging and Graphics*.

[12] Porter AT, Ostrowski MJ (1988). Successful treatment of malignant carcinoid tumour with intravenous Cis-Platinum. *European Journal of Surgical Oncology*.

[13] Timmins PF, Kuo DYS, Anderson PS, Fields AL, Whitney KD, Goldberg GL (2000). Ovarian carcinoid: management of primary and recurrent tumors. *Gynecologic Oncology*.

[14] Knox CD, Feurer ID, Wise PE (2004). Survival and functional quality of life after resection forhepatic carcinoid metastasis. *Journal of Gastrointestinal Surgery*.

[15] Gedaly R, Jeon H, Johnston TD, McHugh PP, Rowland RG, Ranjan D (2008). Surgical treatment of a rare primary renal carcinoid tumor with liver metastasis. *World Journal of Surgical Oncology*.

[16] Landry CS, Scoggins CR, Mcmasters KM, Martin RCG (2008). Management of hepatic metastasis of gastrointestinal carcinoid tumors. *Journal of Surgical Oncology*.

[17] Osborne DA, Zervos EE, Strosberg J (2006). Improved outcome with cytoreduction versus embolization for symptomatic hepatic metastases of carcinoid and neuroendocrine tumors. *Annals of Surgical Oncology*.

[18] Gupta S, Yao JC, Ahrar K (2003). Hepatic artery embolization and chemoembolization for treatment of patients with metastatic carcinoid tumors: the M.D. Anderson experience. *Cancer Journal*.

[19] Strosberg JR, Choi J, Cantor AB, Kvols LK (2006). Selective hepatic artery embolization for treatment of patients with metastatic carcinoid and pancreatic endocrine tumors. *Cancer Control*.

